# A Case Report of Mycobacterium Avium Complex Peritonitis in an AIDS Patient

**DOI:** 10.1155/2013/590478

**Published:** 2013-11-02

**Authors:** Yihenew Negatu, Eyasu Mekonen

**Affiliations:** Department of Medicine, Howard University Hospital, 2041 Georgia Avenue, NW, Washington, DC 20060, USA

## Abstract

Peritonitis due to Mycobacterium avium complex (MAC) infection is uncommon. The risk for MAC in AIDS patients is greatest in those with severely depressed CD4 count. The organs most commonly involved in disseminated MAC infection include spleen, mesenteric lymph nodes, liver, and intestines. The involvement of peritoneum by MAC infection is rare. This is a case of MAC peritonitis in a 26-year-old female AIDS patient who is noncompliant to highly active antiretroviral therapy (HAART). This patient presented with abdominal pain and distension, anorexia, diarrhea, and cough. She was treated with rifabutin, clarithromycin, and ethambutol along with atovaquone for Pneumocystis jiroveci pneumonia prophylaxis and so the patient's condition improved. MAC peritonitis should be considered in a patient presenting with nonspecific abdominal symptoms in the setting of AIDS and low CD4 count.

## 1. Case Report

A 26-year-old female with a history of AIDS who is noncompliant to HAART was admitted for abdominal pain and distension, anorexia, diarrhea, and productive cough of one month duration. She denied fever, night sweats, vomiting, or weight loss.

Physical examination showed pale conjunctivae, clear lung, pedal edema, and no cardiac murmur. Abdominal examination revealed distended abdomen with shifting dullness and diffuse tenderness but no rebound tenderness. No hepatosplenomegaly detected.

**Figure 1:**
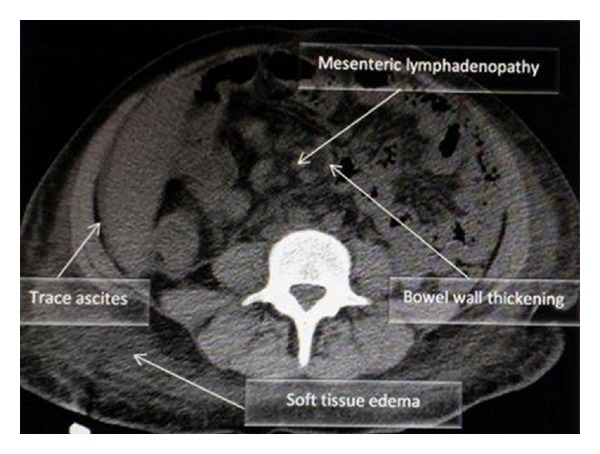
Abdominal CT scan which showed mesenteric lymphadenopathy, bowel wall thickening, trace ascites, and soft tissue edema.

**Figure 2:**
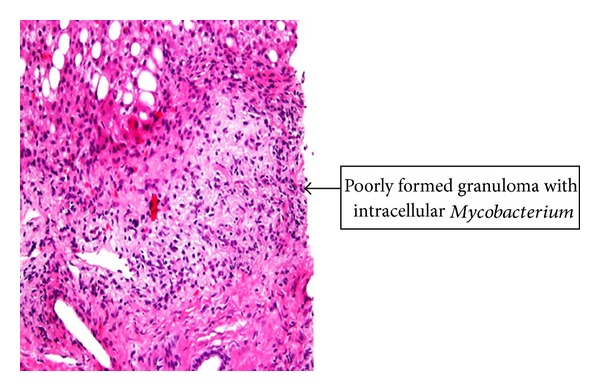
Histopathology of liver biopsy.

**Figure 3:**
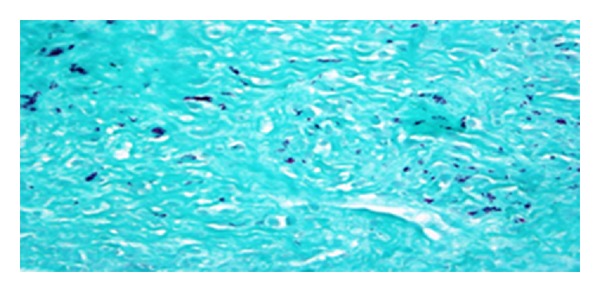
Acid fast stain of the liver biopsy which is positive for AFBs.

Laboratory data showed WBC count of 8800 cells/mm^3^, hematocrit 24.4%, platelets 57,000 cells/mm^3^, CD4 count of 2 cells/mm^3^, ESR 72 mm/hr, total protein 3.9 gm/dL and albumin 1.4 gm/dL, alkaline phosphatase 769 mu/mL, and normal level of transaminases. Peritoneal fluid analysis showed WBC count of 78/mm^3^, predominantly lymphocytes, LDH 102 IU/L, amylase 310 U/L, and albumin <1 g/dL. Chest X-ray showed minimal bilateral effusion with basal atelectasis. CT scan of the abdomen showed moderate ascites, peri-aortic lymphadenopathy, and minimal bowel thickening (see Figure 1). Gram, acid fast, and KOH stains of sputum and ascitic fluid did not show any organism. The cultures of ascitic fluid, peritoneal, and liver biopsies (see Figures 2 and 3), as well as sputum, grew MAC. Patient was started on rifabutin, clarithromycin, and ethambutol for MAC treatment along with atovaquone for Pneumocystis jiroveci pneumonia prophylaxis. The abdominal symptoms and girth showed improvement and patient was discharged with an outpatient clinic appointment for followup and reinitiation of HAART.

## 2. Discussion

MAC infections are caused by either *Mycobacterium avium* or *Mycobacterium intracellulare*. The risk for MAC infection in AIDS patients is greatest in those with severely depressed CD4 count. Disseminated MAC infection is seen rarely in patients with CD4 count of greater than 100 cells/mm^3^ [1]. Although the incidence of MAC infection has declined substantially in the era of HAART, it continues to occur in patients who are noncompliant or without access to HAART or in patients who develop virological and immunological failure to HAART [2]. The organs most commonly involved in disseminated MAC infection include spleen, mesenteric lymph nodes, liver, and intestines [3]. The involvement of peritoneum by MAC infection is rare [1]. There have been only few published case reports on MAC peritonitis. Wu et al. reviewed 16 case reports of MAC peritonitis that were published until 2008. The median age of patients in the review was 36 years and most of them had a CD4 count below 50 cells/*μ*L. Abdominal pain, distension, and diarrhea were the common presenting symptoms [4]. Similarly our patient was a 26-years-old female with CD4 count of 2 cells/mm^3^ and presented with abdominal distention, pain, and diarrhea.

The review indicated that peritonitis was a late manifestation of disseminated MAC infection. Patients had either other sites involved at the time of diagnosis of MAC peritonitis or they had previous history of MAC infection. Our patient also has evidence of involvement of the liver and lymph nodes in addition to the peritoneum. The further dissemination to the peritoneum in this patient may be likely due to noncompliance to antiretroviral treatment. 

The decision to treat this patient with three drug regimen, clarithromycin, ethambutol, and rifabutin, was supported by the study done by the AIDS Clinical Trials Group Study 223 team which demonstrated survival benefit and reduction of risk of relapse as compared to two drugs regimens [5]. 

In conclusion, MAC peritonitis should be considered in patients presenting with non-specific abdominal symptoms in the setting of AIDS and low CD4 count. We believe that this case would serve as a reminder to clinician that even in the era of HAART such rare presentations of MAC infection do occur in AIDS patients.
